# Microbiome Analysis of Stool Samples from African Americans with Colon Polyps

**DOI:** 10.1371/journal.pone.0081352

**Published:** 2013-12-20

**Authors:** Hassan Brim, Shibu Yooseph, Erwin G. Zoetendal, Edward Lee, Manolito Torralbo, Adeyinka O. Laiyemo, Babak Shokrani, Karen Nelson, Hassan Ashktorab

**Affiliations:** 1 Department of Pathology, Department of Medicine and Cancer Center, Howard University, College of Medicine, Washington, District of Columbia, United States of America; 2 JCVI, San Diego, California, United States of America; 3 JCVI, Rockville, Maryland, United States of America; 4 Laboratory of Microbiology, Wageningen University, Wageningen, The Netherlands; University of Illinois, United States of America

## Abstract

**Background:**

Colonic polyps are common tumors occurring in ~50% of Western populations with ~10% risk of malignant progression. Dietary agents have been considered the primary environmental exposure to promote colorectal cancer (CRC) development. However, the colonic mucosa is permanently in contact with the microbiota and its metabolic products including toxins that also have the potential to trigger oncogenic transformation.

**Aim:**

To analyze fecal DNA for microbiota composition and functional potential in African Americans with pre-neoplastic lesions.

**Materials & Methods:**

We analyzed the bacterial composition of stool samples from 6 healthy individuals and 6 patients with colon polyps using 16S ribosomal RNA-based phylogenetic microarray; the Human intestinal Tract Chip (HITChip) and 16S rRNA gene barcoded 454 pyrosequencing. The functional potential was determined by sequence-based metagenomics using 454 pyrosequencing.

**Results:**

Fecal microbiota profiling of samples from the healthy and polyp patients using both a phylogenetic microarraying (HITChip) and barcoded 454 pyrosequencing generated similar results. A distinction between both sets of samples was only obtained when the analysis was performed at the sub-genus level. Most of the species leading to the dissociation were from the *Bacteroides* group. The metagenomic analysis did not reveal major differences in bacterial gene prevalence/abundances between the two groups even when the analysis and comparisons were restricted to available *Bacteroides* genomes.

**Conclusion:**

This study reveals that at the pre-neoplastic stages, there is a trend showing microbiota changes between healthy and colon polyp patients at the sub-genus level. These differences were not reflected at the genome/functions levels. Bacteria and associated functions within the *Bacteroides* group need to be further analyzed and dissected to pinpoint potential actors in the early colon oncogenic transformation in a large sample size.

## Introduction

Colorectal cancer (CRC) is the third most prevalent cancer [1-3]. Its world distribution is heterogeneous with a predominance in the Western world. This heterogeneous distribution is also taking place within the Western societies [4,5]. African Americans (AAs) have a high incidence of, and mortality from this disease [6,7]. Several factors have been proposed and investigated, including genetics, epigenetics, diet, socioeconomic status and access to health [8-15]. However, in comparison with their African counterparts, with whom they share the same genetic background and who have a very low burden of the disease [16], the higher incidence in AAs seems to be caused primarily by environmental factors. 

Emerging data suggest an essential, mutualistic relationship between the host and their colonic microbiota [17-19]. Elegant experiments demonstrated, for example, that a single commensal, *Bacteroides thetaiotamicron* induces colonic mucosal gene expression, angiogenesis and immune responses revealing a broader extent of microbe-mucosal communication and cross-regulation than previously recognized [20]. Similar findings were also obtained with an enterotoxigenic *Bacteroides fragilis* [21,22]. 

Among body sites normally hosting a community of microbes, the human colon harbors the greatest number and diversity of organisms, primarily bacteria. Comprised of 500 to 1,000 bacterial species with two to four million genes, the gut microbiome contains about 100-fold more genes than the human genome and the estimated 10^14^ bacterial cells in the gut exceed by 10-fold the total ensemble of human cells [23]. Molecular analysis of the colonic luminal and mucosal microbiota indicates that individuals harbor unique microbiotas that are fairly stable along the colonic axis. However, the mucosal microbiota is either distinct or contains only a subset of the bacterial phylotypes identified in the luminal fecal samples [24,25]. Although mechanisms accounting for the composition and assemblage of the gut microbiota are incompletely understood, it appears that diet, host genetics, disease state (e.g. obesity, Inflammatory Bowel Disease (IBD)) as well as likely additional environmental factors influence the composition and the function of the colon microbiota [26,27].

The environmental exposures proposed to promote the development of human CRC have been primarily dietary agents. However, the local environment to which the colonic mucosa is exposed is created by the microbiota of the colon and their metabolic products that include beneficial components such as short chain fatty acids as well as harmful ones including toxins. Although it has been hypothesized for decades that the colonic microbiota influence CRC pathogenesis, neither specific bacteria nor mechanisms have been delineated [28-30]. Linkage of specific bacteria, their toxins and/or toxic metabolites (including mutagens) to CRC pathogenesis has been hampered by limited knowledge of the colonic microbiota and changing bacterial classification schemes over the last 40 years. While two divisions of bacteria (*Bacteroidetes* and *Firmicutes*) are considered dominant in the cultured colonic microbiota, up to 80% of the colonic microbiota has not yet been cultured [24,25]. Actinobacteria were also reported as prevalent in the intestinal tract but their presence has been underestimated in PCR based approaches [31]. 

Recent advances in sequencing have set the ground for sequence-based metagenomic studies that target the genomic diversity within an ecosystem Indeed, several studies have set the framework for metagenomic studies in general and for the gut microbiota in particular [24,25,32-36]. Huge databases for 16S rRNA genes as well as for gut microbiota functions have been established as a resource for other studies in the field.

We here performed a microbiome analysis of stool samples from healthy and colon polyp patients to elucidate bacterial changes that might induce or accompany early oncogenic transformation of the colon in African American patients with the goal of defining such markers for non-invasive screening protocols. 

## Materials and Methods

### Ethics statement

The present study was approved by the Howard University Institutional Review Board. Written consent forms were obtained from all participants. 

### Samples collection and preparation

Fecal samples were obtained from 6 healthy (AFR001-006) and 6 colon polyp (AFR007-012) African American individuals who were informed about the study’s goals and consented to participate according to Howard University IRB approved protocol. Patients with family history of colon cancer or inflammatory bowel diseases were excluded from this study. We included in the study adult patients who underwent screening colonoscopy and for whom a pathological diagnosis was established.

Each patient was given a kit with instructions for sample collection in sterile containers. The stool samples were collected at least two months after colonoscopy after a full microbiota restoration [37]. The patients were provided FedEx enveloppes to send samples immediately after bowel movements. The samples were delivered to us on the same day of collection to minimize changes in the microbiota composition. The samples were aliquoted and stored at -80°C. Colonoscopy and pathology reports were reviewed and used to select healthy and colon polyp patients for stool samples collection. The healthy and polyp patients were matched for demographic parameters to reduce the effect of cofounders on microbiota’s differences. All polyp patients have colonic lesions of hyperplastic histology. The Healthy group had a mean age (SD) 59 years (9.4) and a BMI (SD) of 30.8 (4.41) while the polyp patients mean age (SD) was 59 years (8.4) and their BMI (SD) was 31.6 (3.56). There were 3 males and 3 females in both groups that were age matched across the two groups of patients.

### DNA extraction

DNA from the stool samples was extracted using the QIAamp Stool DNA extraction Kit (Qiagen, Inc). The extracted DNA was used for 16S rRNA gene based barcoded 454 pyrosequencing and phylogenetic microarraying for microbiota profiling using the Human Intestinal Tract Chip (HITChip) [38] as well as for the metagenomic analysis.

### HITChip analysis

DNA was used for phylogenetic profiling using the Human Intestinal Tract Chip (HITChip), a phylogenetic microarray which contains a duplicate set of 3,631 probes based on 16S rRNA gene sequences covering more than 1,100 intestinal bacterial phylotypes [38]. Briefly, 20 ng of DNA from each sample (n=12) was used to amplify the nearly full 16S rRNA genes. PCR products were in-vitro transcribed into RNA, labeled with Cy3 and Cy5 and subsequently fragmented. Hybridizations were performed in duplicate and data were extracted from microarray-scanned images using Agilent Feature Extraction software version 10.7.3.1 (http://www.agilent.com). Array normalization was performed using a set of R-based scripts (http://r-project.org) in combination with a custom designed relational database which runs under the MySQL database management system (http://www.mysql.com). 

Hierarchical clustering of probe profiles was carried out by calculating a distance matrix between the samples based on the squared difference between each pair of profiles (Euclidian distance). The distance matrix was used in the hclust implementation in R of a hierarchical clustering algorithm. The agglomeration method used in this algorithm was Ward’s minimum variance method. The bacterial composition was compared at the phylum level (divided into class level for the *Firmicutes*) and at the genus-like level (131 phylogenetic groups with 90% or more 16S rRNA gene sequence similarity) using the Wilcoxon signed-rank test that was corrected for multiple comparisons (q value), in which q<0.05 was considered significantly different. The diversity was determined calculated using the Shannon index of diversity on probe signal intensities. Principle component analysis based on probe profiles was performed using CANOCO 4.5 software package (Biometrics, Wageningen, the Netherlands).

### 16S rRNA profiling by 454 pyrosequencing

DNA from each of the 12 stool samples was amplified using primers that targeted the V1-V3 regions of the 16S rRNA gene [39]. These primers included the A and B adaptor sequences for 454 pyrosequencing as well as a unique 12 bp barcode incorporated onto the reverse primer such that each sample receives its own unique barcode. The barcode sequences for all 12 samples are provided in File S2. This method of incorporating the A and B adaptors onto the primers at the PCR stage provided minimal loss of sequence data when compared to previous methods that would ligate the A and B adaptors to every amplicon after amplification. This method also allows to generate sequence reads which are all in the same 5’-3’ orientation. Using approximately 100ng of extracted DNA, the amplicons were generated with Platinum Taq polymerase (Invitrogen, CA) and by using the following cycling conditions: 95°C for 5min for an initial denaturing step followed by 95°C for 30 sec, 55°C for 30 sec, 72°C for 30 sec for a total of 35 cycles followed by a final extension step of 72°C for 7 min then stored at 4°C. Once the PCR for each sample was completed, the amplicons were purified using the QIAquick PCR purification kit (Qiagen Valencia, CA), quantified, normalized, and then pooled in preparation for emulsion PCR followed by 454 sequencing using Titanium chemistry (Roche, Basel Switzerland) following the manufacturer’s protocol. In the first step of data processing, the generated sequence data were deconvolved using the sample barcodes to identify sequences from each of the samples. Barcode, primer, and adaptor sequences were also trimmed as part of this step. PCR artifacts “chimeras” were identified using the ChimeraSlayer program (http://microbiomeutil.sourceforge.net; reference http://genome.cshlp.org/content/21/3/494.long), and removed prior to downstream analysis. The resulting deconvoluted and filtered sequence data were assigned taxonomy (to the genus level) using the Ribosomal Database Project (RDP) classifier [40] and the genera classifications were used to generate a sample-genus count matrix. Operational Taxonomic Unit (OTU) analysis of these sequences was performed as follows: sequences were processed (trimmed) using the Mothur software [41] and subsequently clustered at 97% sequence identity [42] using cd-hit [43] to generate OTUs. The OTU memberships of the sequences were used to construct a sample-OTU count matrix. The samples were clustered at genus and OTU levels using the sample-genus and sample-OTU count matrices respectively. For each clustering, Morisita-Horn dissimilarity was used to compute a sample distance matrix from the initial count matrix, and the distance matrix was subsequently used to generate a hierarchical clustering using Ward’s minimum variance method. The Wilcoxon Rank Sum test was used to identify OTUs that had differential abundance in the healthy and polyp sample groups.

### Metagenomic analysis

Prepared DNA from the 12 stool samples was processed using the Genomiphi MDA kit (GE-Healthcare) using the manufacturer’s suggested protocol, in preparation for library construction for 454 sequencing.  The sample preparation process in this system involves fragmentation of MDA amplified genomic DNA, followed by ligation of MID barcodes and 454 adaptor sequences.   Each sample was then normalized, pooled then loaded into a half picoliter plate for 454 sequencing.  The samples were then amplified using emulsion PCR followed by 454 sequencing using Titanium chemistry. The metagenomic sequence data were first processed to remove 454 artifacts (replicate reads arising from the emulsion PCR process) [44] and then genes were identified on reads using a frameshift tolerant gene finder (FragGeneScan) [45] so as to overcome any 454 homopolymer problems. These genes were then searched against the KEGG database to identify kegg ortholog counts [46,47].

All of the 16S and metagenomic data generated in this study have been deposited in the NCBI's Sequence Read Archive (http://www.ncbi.nlm.nih.gov/bioproject/222611).

## Results

### HITChip based taxonomy analysis

**Figure 1:**
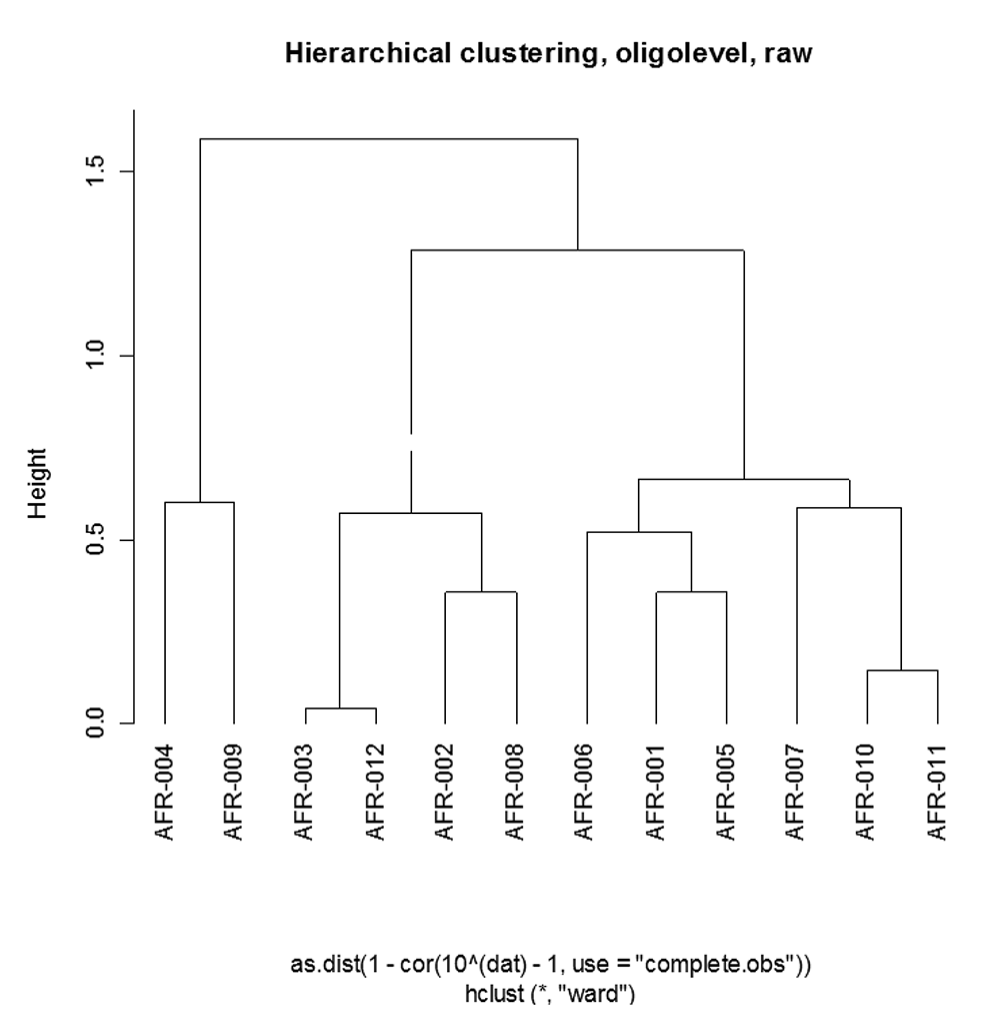
Dendrogram of the samples’ clustering based on the global HITChip oligonucleotide probe signal intensities (AFR001-006; Healthy patients’ samples, AFR007-012: Polyp patients’ samples).

The HITChip analysis revealed the bacterial profile within each of the analyzed samples as well as the clustering of these samples based on profiles’ similarities/dissimilarities. The clustering based on the generated profiles did not reveal a clear separation between the polyp and healthy samples’ profiles. The samples in the generated clustering were intermixed with 3 polyp (AFR011, 010 & 007) and 3 healthy samples (AFR005, 001 & 006) on one side of the dendrogram located distantly from the other polyp (AFR008, 009 & 012) and healthy samples (AFR002, 003 & 004) (Figure 1). 

**Figure 2:**
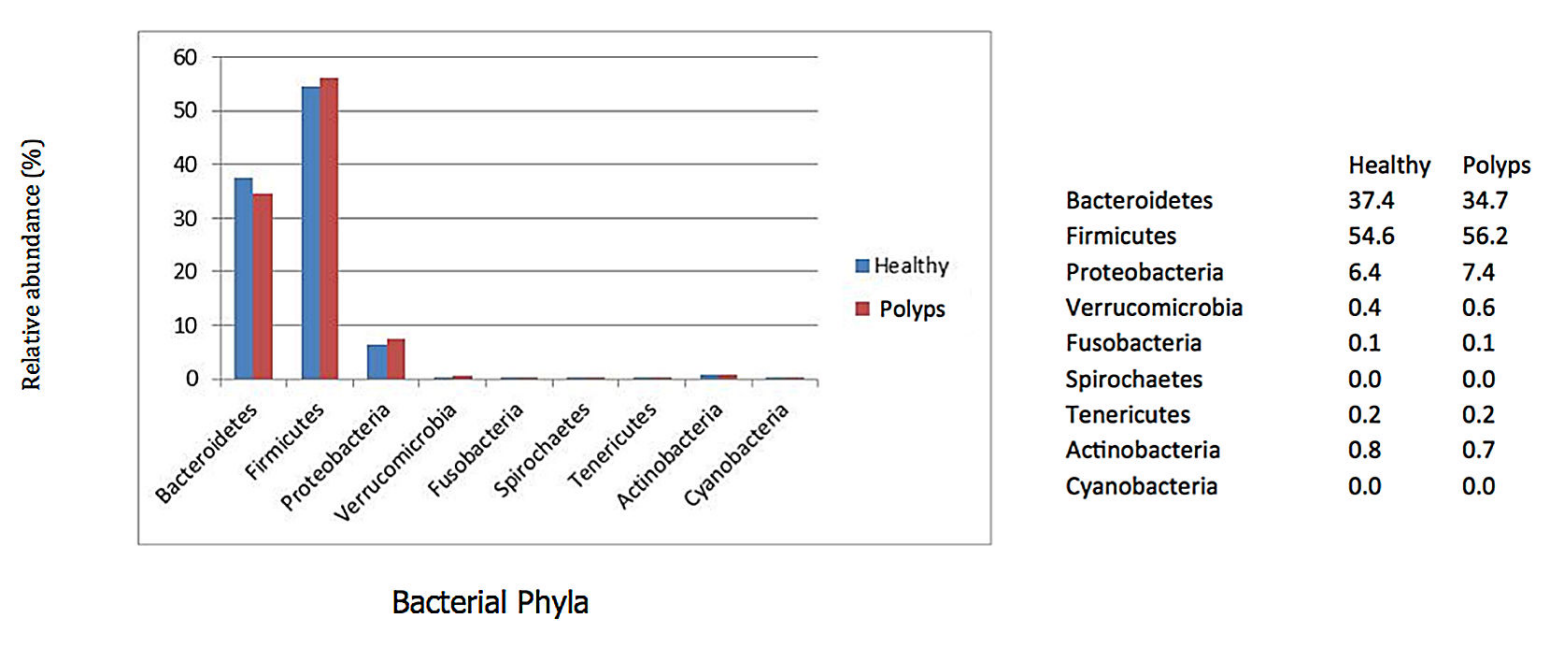
Barplots showing the relative abundances of bacterial phyla in healthy and colon polyps patients’ samples determined by HITChip profiling.

**Figure 3:**
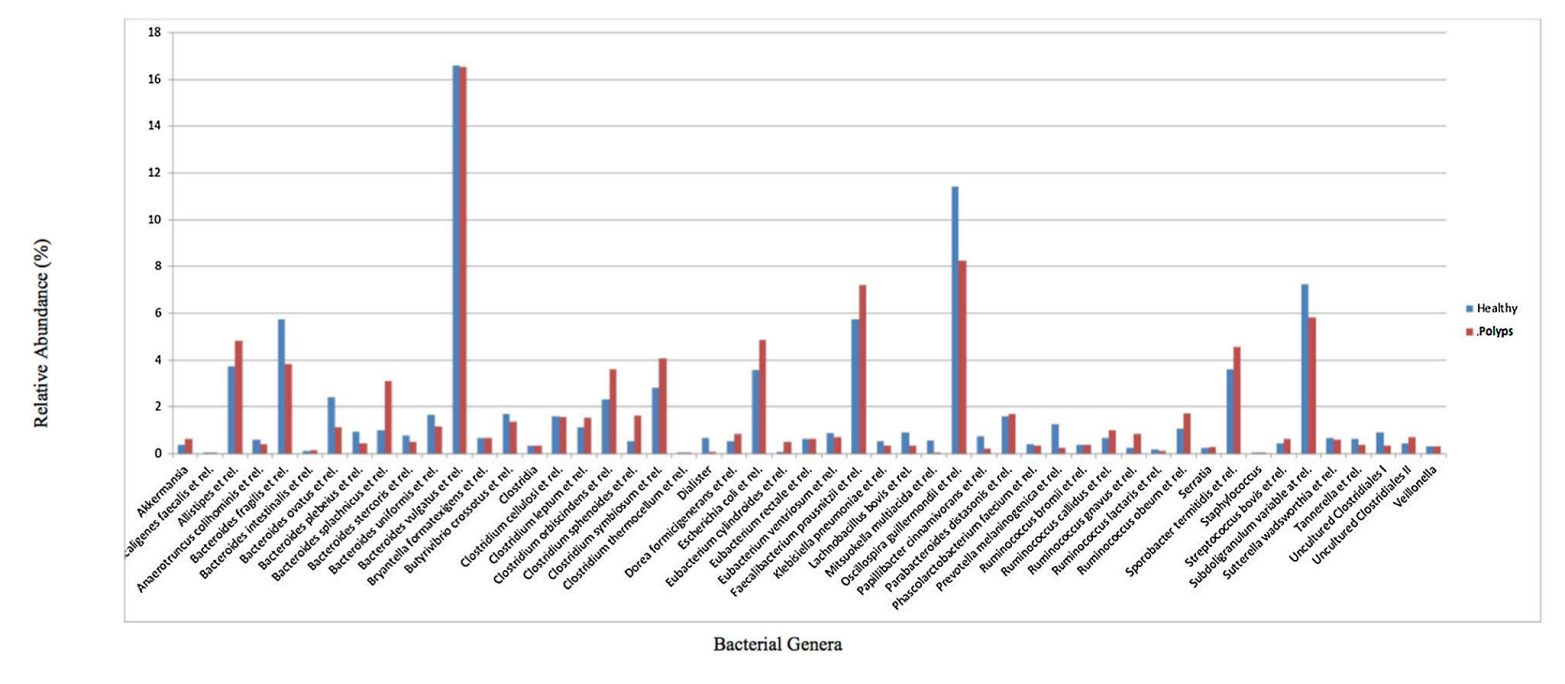
Barplots showing the relative abundances of bacterial groups at genus-like level in healthy and colon polyps patients’ samples determined by HITChip profiling. Bacterial groups that are at least 1% in one of the samples are shown.

*Bacteroidetes* and *Firmicutes* were prevalent in all samples and totaling about 92% of the total detected bacteria. Another important group was Proteobacteria which was represented by ~7% of the probes in both sets of samples. Healthy individuals had a relatively higher prevalence of *Bacteroides* (37.4 vs. 34.7%) while polyp samples had a higher prevalence of *Firmicutes* (56.2 vs. 54.6%). The prevalence of Proteobacteria was 6.4 and 7.4 in healthy and polyp samples, respectively (Figure 2). At the genus level, there were several bacteria that displayed relative abundances in the analyzed sets of samples as depicted in Figure 3 and associated File S1. 

### 454 pyrosequencing taxonomic analysis

**Figure 4:**
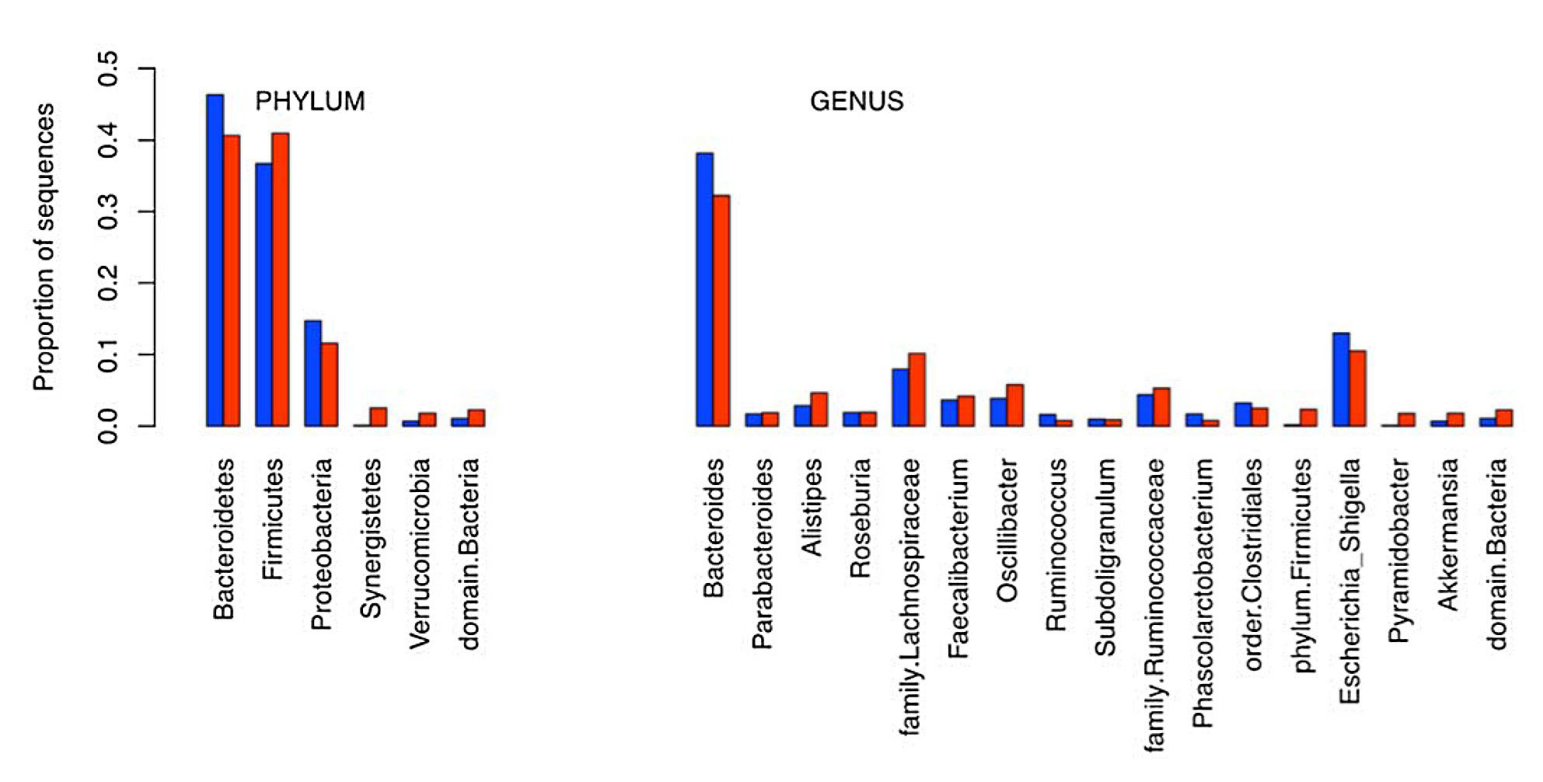
Bacterial phyla and genera distribution in healthy (Blue) and colon polyps (Red) patients’ samples based on 454 pyrosequencing data.

**Figure 5:**
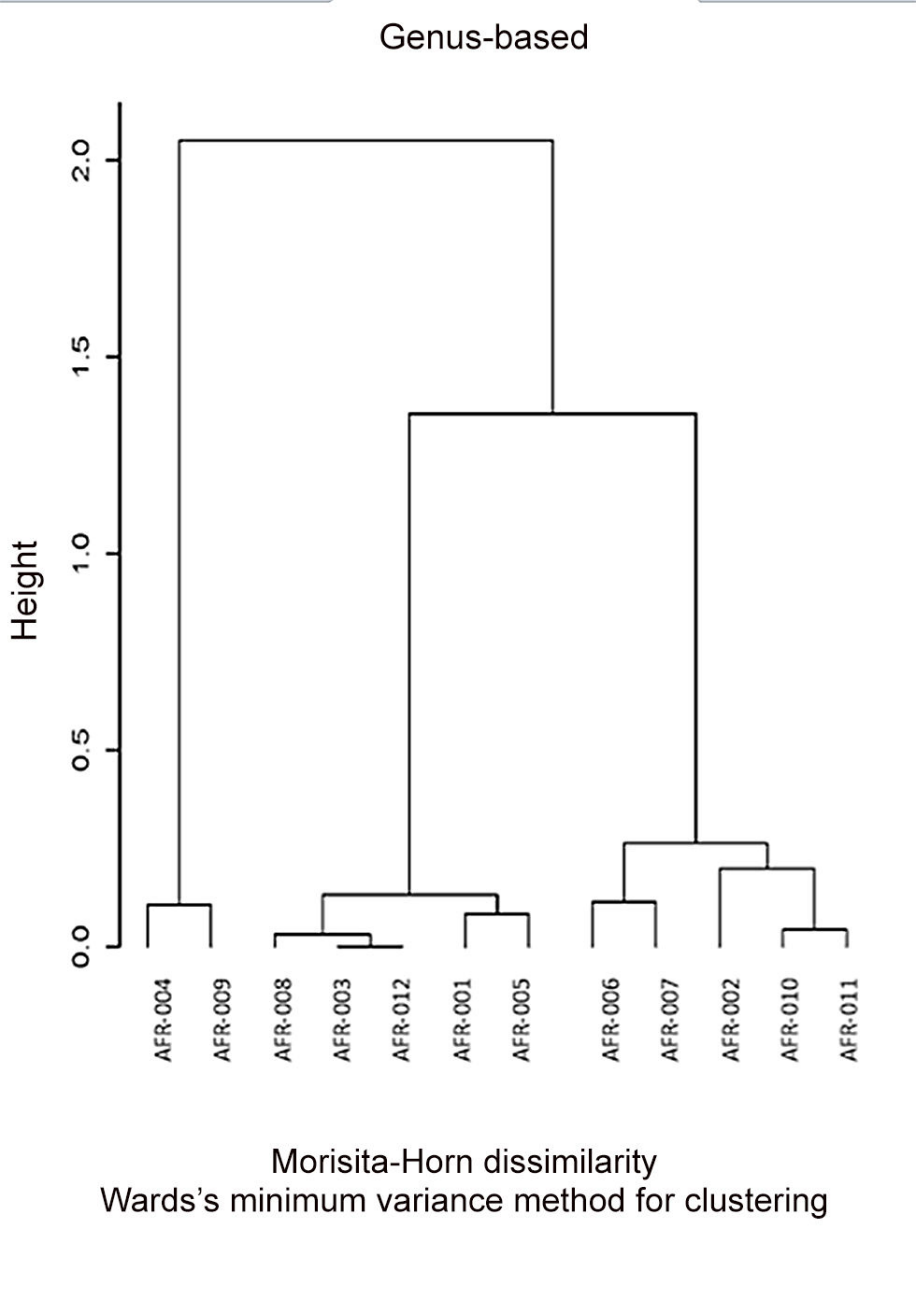
Dendrogram reflecting the healthy (AFR001-006) and colon polyps (AFR007-012) samples clustering based on the 454 pyrosequencing data analyzed at the genus level.

In parallel to the HIT Chip analysis, we analyzed the same sets of samples using 454 pyrosequencing where V1-V3 variable region of the 16S rDNA sequence was PCR amplified and sequenced. The generated sequences were analyzed using the Ribosomal Database project data and sequence identification was established (File S2). *Bacteroides* and *Firmicutes* represented 80 to 85% of the sequences in both sets of samples with a higher prevalence of *Bacteroides* in healthy samples vs polyps and a higher prevalence of *Firmicutes* in polyp samples vs. healthy ones. Proteobacteria were the third major group of bacteria accounting for ~10% of the sequences (Figure 4). A clustering of the samples based on the generated sequences at the genus level led to the dendrogram depicted in Figure 5, in which the healthy and polyp samples were intermixed. Polyp samples AFR011, 010 & 007 clustered with healthy samples AFR002 & 006 while polyp samples AFR008, 009 & 012 clustered with healthy samples AFR001, 003, 004 and 005 (Figure 5).

**Figure 6:**
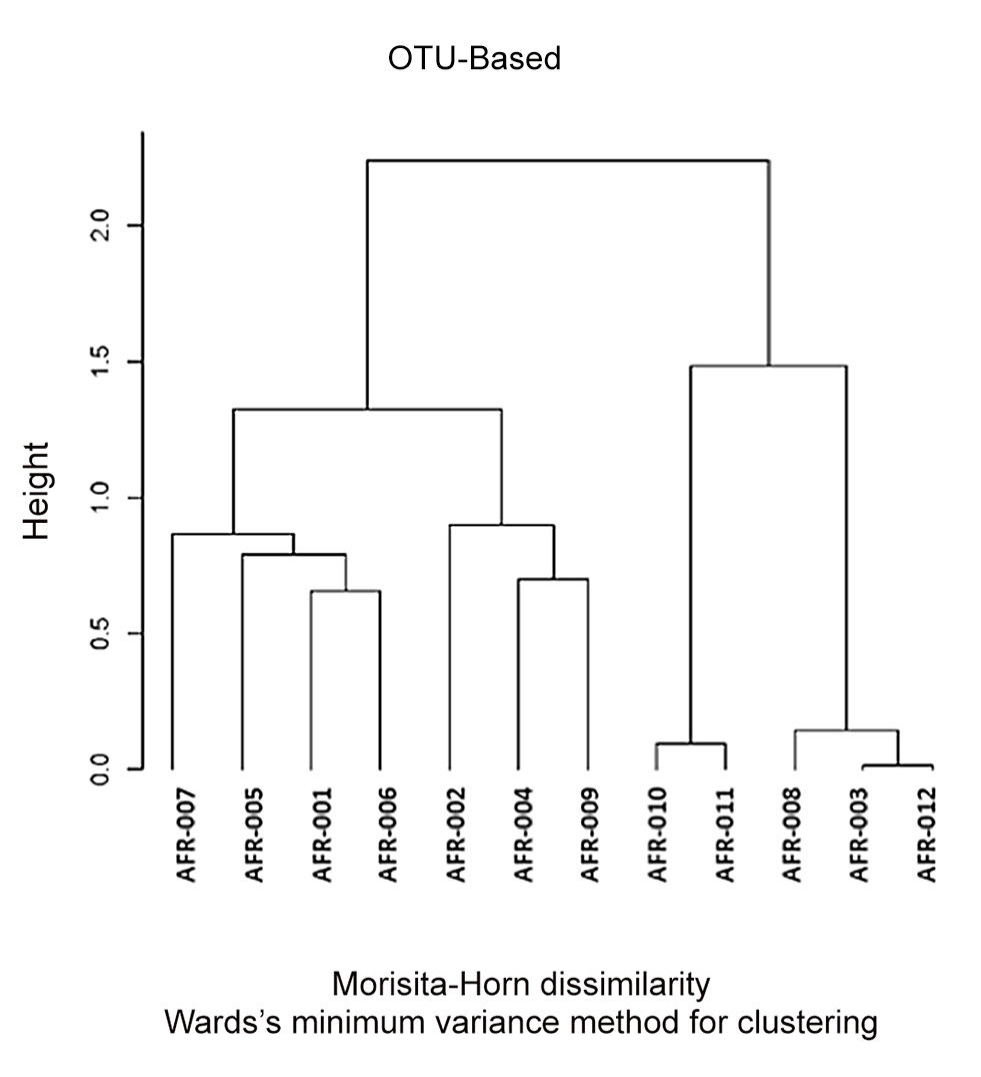
Dendrogram reflecting the healthy (AFR001-006) and colon polyps (AFR007-012) samples clustering based on the 454 pyrosequencing data analyzed at the Operational Taxonomic Unit (OTU: sub-genus) level.

Further clustering at the Operational Taxonomic Units level (OTU: sub-genus level) led to a different repartition of the analyzed samples (Figure 6). In this figure, a better resolution was obtained with polyp samples AFR008, 010, 011 & 012 clustering together with healthy sample AFR003 while polyp samples 006 and 007 clustered with the other healthy samples (Figure 6). More importantly, the height (branching distance) obtained at the sub-genus comparison (Figure 6) was bigger than that obtained in the genus level comparison (Figure 5) pointing to much more differences between the two groups at the OTU rather than the genus level. Seven out of 11 OTUs that led to this separation consisted of *Bacteroides* (Table 1).

**Table 1:** Differential OTUs leading to the separate clustering of the healthy and colon polyps samples the sub-genus level data analysis.

•OTU	p-value	Taxonomy					
•OTU_1776	0.002165	Bacteria	Bacteroidetes	Bacteroidia	Bacteroidales	Bacteroidaceae	Bacteroides
•OTU_1807	0.002165	Bacteria	Firmicutes	Clostridia	Clostridiales	Lachnospiraceae	
•OTU_96	0.016059	Bacteria	Bacteroidetes	Bacteroidia	Bacteroidales	Bacteroidaceae	Bacteroides
•OTU_1775	0.016711	Bacteria	Bacteroidetes	Bacteroidia	Bacteroidales	Bacteroidaceae	Bacteroides
•OTU_338	0.028441	Bacteria	Bacteroidetes	Bacteroidia	Bacteroidales	Bacteroidaceae	Bacteroides
•OTU_485	0.030348	Bacteria	Firmicutes	Clostridia	Clostridiales	Lachnospiraceae	
•OTU_1312	0.037941	Bacteria	Bacteroidetes	Bacteroidia	Bacteroidales	Bacteroidaceae	Bacteroides
•OTU_224	0.037941	Bacteria	Firmicutes	Clostridia	Clostridiales		
•OTU_1127	0.040253	Bacteria	Firmicutes	Erysipelotrichi	Erysipelotrichales	Erysipelotrichaceae	Coprobacillus
•OTU_1554	0.041596	Bacteria	Bacteroidetes	Bacteroidia	Bacteroidales	Bacteroidaceae	Bacteroides
OTU_177	0.043826	Bacteria	Bacteroidetes	Bacteroidia	Bacteroidales	Bacteroidaceae	Bacteroide

### Metagenomic analysis

The raw count for Kegg Orthologs (KOs) were used to calculate the proportion of KOs in each of the twelve metagenomic datasets (Files S2, S3 & S4) using a method that accounts for difference in gene lengths [46,47]. These proportions were used in a Wilcoxon Rank Sum test to identify kegg orthologs that were differentially abundant in the healthy and disease sample groups. Overall, after correcting for multiple testing, we did not see statistically significant groups (Kegg Orthologs) using a false discovery rate of 5%. Because most of the OTUs leading to a higher resolution of the analyzed samples were from the *Bacteroides* group, we performed an analysis of the metagenomic data against available *Bacteroides* genomes (File S5). All reads from each sample were searched against available *Bacteroides* genomes. A read was considered as mapping to a genome if its match to the genome had ≥80% identity and covered ≥80% of the read sequence. Overall, the healthy samples have a slightly higher mean proportion of recruitment to *Bacteroides*, though this seems to be driven primarily by sample AFR005. The IDs of all *Bacteroides* genome sequences used in this analysis are included in File S5. 

## Discussion

The microbiota have long been overlooked and nowadays there is an increasing interest in studies that seek to define their role in health and disease [48-50]. The most important site for these studies is the gut since the intestinal microbiota plays major roles in nutrition, metabolism and immunity [48-50]. It has been well documented that many intestinal diseases have bacterial components such as in the case of ulcerative colitis and Crohn’s disease [51]. Their role in triggering or promoting colon oncogenic transformation has yet to be established even though many publications reported the potential of many individual bacteria to induce tumorigenesis in germ free mice [21,22].

Here we report experiments where we show that there is a trend of specific bacterial profiles in colon polyp patients when compared to healthy individuals. We used two technologies to investigate the microbiota’s profiles in our sets of samples. Both the phylogenetic microarraying using the HITChip and 454 pyrosequencing generated similar results and similar bacterial groups distribution. Similar findings were reported by Van den Bogert et al. who reported comparable results from stool and small intestinal samples when the two technologies were compared even when different sets of 16S rRNA primers were used [52]. 

The genus based clustering using data from both technologies was similar which reflects the strength of both technologies in microbiota profiling. However, no clear resolution of the two sets of samples was obtained at the genus level. Further analysis at the sub-genus level, led to much clearer separation of the samples with 4 of the polyp patients bacterial profiles clustering on one side of the generated dendrogram with bigger branching distance separating the two clusters in the sub-genus dendrogram (Figures 5 & 6). This finding is to be expected since changes in the microbiota in the oncogenic transformation are thought to be taking place within the existing microbiota where previously less represented bacterial strains become dominant or unexpressed bacterial functions become induced in response to some environmental stressors [53,54]. Diet is known to be an important effector of both microbiota composition and colorectal cancer risk. The magnitude and nature of its effects can only be assessed in well controlled prospective studies. 

*Bacteroidetes* and *Firmicutes* were the most prevalent groups of bacteria detected in all samples. Proteobacteria corresponded to the next group of relevance in our samples. 

The sub-genus bacteria that led to the new clustering were predominantly from the *Bacteroides* group in the polyp patients’ samples (7 out of 11). Yoshino et al. have reported that *Bacteroides* bacteremia as strongly associated with colorectal cancer in Japanese patients [55]. Also, Wu et al. have shown that strains from the *Bacteroides fragilis* species carrying the bft gene (toxin) are able to promote colon oncogenic transformation in mice models of colon cancer through pSTAT3 pathway [21,22]. Also, Toprak et al. did report a 38% prevalence of bft in colon cancer stool samples vs. 12% in control patients [56]. 

In an immunohistochemistry experiment using a pSTAT3 antibody on tissue microarray using both African American normal and adenoma tissue samples, adenoma samples were strongly stained when compared to normal samples (data not shown). pSTAT3 is the preferential pathway induced by bft toxin and other bacterial antigens [57]. While other bacterial toxins, from *Bacteroides* bacteria or others, might trigger the pSTAT3 pathway as well, our results do still point to the need to further dissect such functions in *Bacteroides* strains.

The metagenomic analysis in our study was more descriptive of the potential of the microbiome in African American microbiota but did not reveal any statistically significant markers or functions when the samples were compared either individually or as two groups. Overall, the usual bacterial functions were detected in the analyzed samples (See Files S2, S3 & S4). A second analysis of the metagenomic data was done in comparison to all known *Bacteroides* genomes (n=90). This analysis did not lead to significant differences between the two sets of samples as well. 

It is noteworthy that recent publications have reported the prevalence of *Fusbacterium* spp and *Fusobacterium nucleatus* [58-60] in colon cancer tumors when compared to normal colon samples. Such was not the case in the analyzed stool samples. This finding points probably to the importance of analyzing and establishing bacterial markers of colon oncogenic transformation in colon tissues and subsequent validation in stool samples. Indeed, adherent bacteria might be more prone to affect gene expression in colon mucosal cells than transient bacteria that are flushed in the fecal samples. Large studies of stool and colon tissue samples from different stages of colon cancer development are needed to establish strong bacterial markers of oncogenic transformation.

## Supporting Information

File S1 **HITChip bacterial genera distribution in the analyzed samples.**
(DOCX)

File S2 **454 Pyrosequencing barcodes and data for all samples.**
(XLS)

File S3 **Number of metagenomic reads analyzed for all 12 samples.**
(XLSX)

File S4 **KEGG analysis of the metagenomic data in the two sets of samples.**
(XLSX)

File S5 ***Bacteroides* strains against which genomes the metagenomic data was analyzed.**
(XLSX)
