# Supplementary material for: Microbiome Analysis of Stool Samples from African Americans with Colon Polyps
Source: PLoS One. 2013 Dec 20;8(12):e81352. doi: 10.1371/journal.pone.0081352 (PMC3869648; doi:10.1371/journal.pone.0081352)
Supplement: File S1 — HITChip bacterial genera distribution in the analyzed samples. (DOCX) [file pone.0081352.s001.docx]

File S1: HITChip bacterial genera distribution in the analyzed samples

|  | Healthy | Polyps |
| --- | --- | --- |
| Akkermansia | 0.39 | 0.63 |
| Alcaligenes faecalis et rel. | 0.05 | 0.05 |
| Allistipes et rel. | 3.75 | 4.84 |
| Anaerotruncus colihominis et rel. | 0.62 | 0.41 |
| Bacteroides fragilis et rel. | 5.76 | 3.85 |
| Bacteroides intestinalis et rel. | 0.12 | 0.14 |
| Bacteroides ovatus et rel. | 2.43 | 1.15 |
| Bacteroides plebeius et rel. | 0.93 | 0.44 |
| Bacteroides splachnicus et rel. | 1.01 | 3.12 |
| Bacteroides stercoris et rel. | 0.76 | 0.50 |
| Bacteroides uniformis et rel. | 1.68 | 1.16 |
| Bacteroides vulgatus et rel. | 16.61 | 16.54 |
| Bryantella formatexigens et rel. | 0.67 | 0.67 |
| Butyrivibrio crossotus et rel. | 1.70 | 1.37 |
| Clostridia | 0.35 | 0.34 |
| Clostridium cellulosi et rel. | 1.60 | 1.57 |
| Clostridium leptum et rel. | 1.15 | 1.53 |
| Clostridium orbiscindens et rel. | 2.31 | 3.60 |
| Clostridium sphenoides et rel. | 0.54 | 1.62 |
| Clostridium symbiosum et rel. | 2.83 | 4.05 |
| Clostridium thermocellum et rel. | 0.01 | 0.01 |
| Dialister | 0.66 | 0.10 |
| Dorea formicigenerans et rel. | 0.54 | 0.84 |
| Escherichia coli et rel. | 3.57 | 4.87 |
| Eubacterium cylindroides et rel. | 0.06 | 0.52 |
| Eubacterium rectale et rel. | 0.66 | 0.63 |
| Eubacterium ventriosum et rel. | 0.87 | 0.69 |
| Faecalibacterium prausnitzii et rel. | 5.77 | 7.22 |
| Klebisiella pneumoniae et rel. | 0.55 | 0.33 |
| Lachnobacillus bovis et rel. | 0.90 | 0.34 |
| Mitsuokella multiacida et rel. | 0.57 | 0.04 |
| Oscillospira guillermondii et rel. | 11.43 | 8.27 |
| Papillibacter cinnamivorans et rel. | 0.74 | 0.21 |
| Parabacteroides distasonis et rel. | 1.60 | 1.70 |
| Phascolarctobacterium faecium et rel. | 0.40 | 0.34 |
| Prevotella melaninogenica et rel. | 1.26 | 0.23 |
| Ruminococcus bromii et rel. | 0.39 | 0.39 |
| Ruminococcus callidus et rel. | 0.67 | 1.02 |
| Ruminococcus gnavus et rel. | 0.23 | 0.83 |
| Ruminococcus lactaris et rel. | 0.17 | 0.10 |
| Ruminococcus obeum et rel. | 1.07 | 1.74 |
| Serratia | 0.25 | 0.29 |
| Sporobacter termitidis et rel. | 3.62 | 4.56 |
| Staphylococcus | 0.03 | 0.03 |
| Streptococcus bovis et rel. | 0.45 | 0.64 |
| Subdoligranulum variable at rel. | 7.23 | 5.80 |
| Sutterella wadsworthia et rel. | 0.66 | 0.61 |
| Tannerella et rel. | 0.63 | 0.38 |
| Uncultured Clostridiales I | 0.91 | 0.36 |
| Uncultured Clostridiales II | 0.43 | 0.71 |
| Veillonella | 0.30 | 0.31 |
